# Emission of circularly polarized light by a linear dipole

**DOI:** 10.1126/sciadv.aav7588

**Published:** 2019-06-28

**Authors:** Martin Neugebauer, Peter Banzer, Sergey Nechayev

**Affiliations:** 1 Max Planck Institute for the Science of Light, Staudtstr. 2, D-91058 Erlangen, Germany.; 2 Institute of Optics, Information and Photonics, University Erlangen-Nuremberg, Staudtstr. 7/B2, D-91058 Erlangen, Germany.

## Abstract

Controlling the polarization state and the propagation direction of photons is a fundamental prerequisite for many nanophotonic devices and a precursor for future on-chip communication, where the emission properties of individual emitters are particularly relevant. Here, we report on the emission of partially circularly polarized photons by a linear dipole. The underlying effect is linked to the near-field part of the angular spectrum of the dipole, and it occurs in any type of linear dipole emitter, ranging from atoms and quantum dots to molecules and dipole-like antennas. We experimentally observe it by near-field to far-field transformation at a planar dielectric interface and numerically demonstrate the utility of this phenomenon by coupling the circularly polarized light to the individual paths of crossing waveguides.

## Teaser

We show that an individual linear dipole emitter—contrary to common belief—can also radiate circularly polarized light.

## INTRODUCTION

Although dipole emitters represent the most fundamental and well-understood sources of photons, recent studies still reveal peculiar effects in their emission characteristics. For instance, different combinations of electric and/or magnetic dipole moments can result in highly directional far-field emission (*1*), spin segmentation (*2*, *3*), well-defined far-field helicities (*4*, *5*), and directional near-field coupling (*6*, *7*). These findings led to various applications in nano-optical experiments, ranging from single-atom optical isolators (*8*, *9*) and deterministic single-photon waveguide couplers (*10*) to position sensing (*11*), polarization-dependent switching (*12*), chirality enhancement (*13*), and the design of novel meta-surfaces (*14*), to name a few.

In this letter, we investigate the angular spectrum of a linear electric dipole and reveal the occurrence of partially circularly polarized light in the evanescent (nonpropagating near-field) part of the *k* space. This circular polarization can be separated into two different components: fields spinning transverse with respect to the propagation direction of the individual evanescent waves—an effect referred to as transverse spin that has been intensely investigated in recent years (*15*–*21*)—and fields spinning around the propagation direction similar to circular polarization in paraxial beams of light, which we refer to as longitudinal spin (*17*). For the demonstration of the latter, we experimentally couple the longitudinal spin of the near field to the far field—where it can be observed as circularly polarized light—by introducing an optically denser medium in close proximity to the dipole. Furthermore, we elaborate on the practical implications of this circularly polarized emission in a waveguide coupling arrangement.

## RESULTS

### Theoretical description of a linear dipole

The electric field **E**(**r**) radiated by an oscillating monochromatic dipole moment **p** located at the origin of the Cartesian coordinate system can be written in SI units as (*22*)

$$E(r)=\frac{\text{exp}({\mathrm{ik}}_{0}r)}{4{\pi \varepsilon }_{0}r}k_{0}^{2}\{({\hat{e}}_{r}\times p)\times {\hat{e}}_{r}+[3{\hat{e}}_{r}({\hat{e}}_{r}\cdot p)-p](\frac{1}{k_{0}^{2}r^{2}}-\frac{i}{k_{0}r})\}$$ (1)

with the wave number in free-space *k*_0_ = 2π/λ and wavelength λ. The unit vector ${\hat{e}}_{r}$ points away from the dipole in radial direction and ε_0_ is the vacuum permittivity. Alternatively, **E**(**r**) can be represented as a superposition of all plane waves launched by the dipole. The local field is thereby given by a Fourier integral (*23*)

$$E(r)\propto \iint \tilde{E}(k)e^{i[k_{x}x+k_{y}y+k_{z}|z|]}\;{\mathrm{dk}}_{x}\;{\mathrm{dk}}_{y}$$ (2)

$\tilde{E}(k)$ is called the vectorial angular spectrum (VAS) of the dipole. The VAS of a linear dipole—here we consider a dipole parallel to the *x* direction—can be written as (*23*, *24*)

$$\tilde{E}(k)\propto \frac{1}{k_{z}}(\begin{array}{c} \frac{k_{0}^{2}-k_{x}^{2}}{k_{0}^{2}}\\ -\frac{k_{x}k_{y}}{k_{0}^{2}}\\ \mp \frac{k_{x}k_{z}}{k_{0}^{2}} \end{array})$$ (3)

This is the *k*-space representation of the dipole field with respect to the *x*-*y* plane in real space (*k_x_*-*k_y_* plane in *k* space). As a practical notation, we define the transverse *k* vector as **k**_⊥_ = (*k_x_*, *k_y_*,0) and the longitudinal component by $k_{z}={\left(k_{0}^{2}-k_{⊥}^{2}\right)}^{1/2}$ with imaginary part Im (*k_z_*) ≥ 0. The ∓ sign in Eq. 3 refers to regions with *z* ≷ 0, respectively.

We are specifically interested in the circularly polarized emission or, equivalently, the spin of the emitted light. In complex three-dimensional field distributions, the spin density describes the local orientation and strength of the circular polarization. For real and *k* space, we can use similar definitions (*18*–*20*)

$$s(r)=\text{Im}\;(E*\times E),\;\tilde{s}(k)=\text{Im}\;(\tilde{E}*\times \tilde{E})$$ (4)

where we omit all prefactors for the sake of simplicity. Regarding the VAS, we distinguish two different regions in *k* space. First, the propagating part of the VAS defined by ∣**k**_⊥_∣ ≤ *k*_0_ and *k_z_* ∈ ℝ. In this angular region, all components of the polarization vector in Eq. 3 are real, and $\tilde{s}=0$. Second, for ∣**k**_⊥_∣ > *k*_0_, we result in an imaginary *k_z_* = *i*∣*k_z_*∣ (from now on, we consider the half space with *z* > 0), indicating the evanescent part of the VAS. Concerning the polarization vector in Eq. 3, only the *z* component contains *k_z_*, which implies that $\tilde{E_{z}}$ is phase-shifted with respect to $\tilde{E_{x}}$ and $\tilde{E_{y}}$ by ±π/2, giving rise to a certain degree of circular polarization. To investigate the orientation of the polarization ellipse, we decompose the spin density **s** into components parallel (${\tilde{s}}_{k}$) and transverse (${\tilde{s}}_{t}$) with respect to the propagation direction defined as the real part of the *k* vector, *Re*(**k**) = **k**_⊥_ (details can be found in Materials and Methods). In our representation, ${\tilde{s}}_{k}$ describes the electric field vector spinning around the propagation direction, similar to the circular polarization of paraxial beams of light (*18*–*20*). In contrast, ${\tilde{s}}_{t}$ describes fields that spin around a transverse axis, similar to the spokes of a wheel spinning around an axis perpendicular to the propagation direction of the wheel (*20*).

**Fig. 1:**
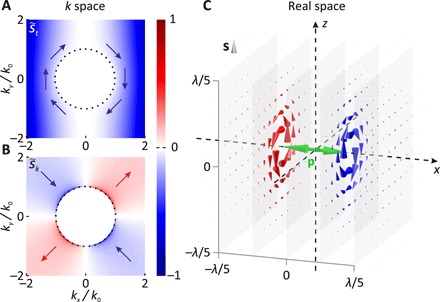
The spin angular momentum density of a linear dipole in *k* space and in real space. (**A**) Transverse and (**B**) longitudinal spin density components (${\tilde{s}}_{t}$ and ${\tilde{s}}_{k}$) of the VAS of a linear dipole moment oriented along the *x* axis. The dotted black circles mark the transition from the propagating to the evanescent part of the VAS, and the red and blue arrows indicate the local orientation of the corresponding spin density components. Both spin density components are normalized to the same value. (**C**) Real-space spin density distribution **s** in the near field of a linear dipole (dipole moment orientation along the *x* axis indicated by the green vector). The spin density is plotted as cones for five planes of observation marked in gray. The red and blue colors highlight counterclockwise and clockwise distributions of the spin density, respectively.

We plot both components of the spin density of the VAS in Fig. 1 (A and B). As already mentioned, in the propagating region of the *k* space—within the dotted black circles—the spin density is zero in all its components. However, in the evanescent part of the VAS (∣**k**_⊥_∣ > *k*_0_), the transverse ${\tilde{s}}_{t}$ shows a two-lobe pattern, and the maximum amplitude occurs along the *x* axis. This transverse spin occurring in evanescent waves is well understood and has been investigated in many scenarios (*18*–*20*). Besides the transverse component, we additionally observe a nonzero longitudinal component ${\tilde{s}}_{k}$, which is strongest along the ±45° directions, exhibiting a four-lobe pattern with alternating sign (a geometrical model describing the origin of the distribution of ${\tilde{s}}_{k}$ can be found in the Supplementary Materials). The occurrence of ${\tilde{s}}_{k}$ is astonishing because the real-space field distribution of a linear dipole does not show any spin being parallel to the propagation direction of the spherical wave. For demonstration, we depict the spin density distributions surrounding a linear dipole in real space for several planes of observation (Fig. 1C). As we can see, the spin density indicated by red- and blue-colored cones exhibits a purely transverse, azimuthal distribution around the axis of the linear dipole moment **p**, which is marked by the green vector. No longitudinal spin component (this is a spin component pointing away from the dipole in radial direction ${\hat{e}}_{r}$) occurs and $s{\hat{e}}_{r}=0$ [further discussion on **s**(**r**) and its derivation is provided in the Supplementary Materials]. That is, knowing that the evanescent part of the VAS dominates the near field in real space (*24*), although their relation is not straightforward (*23*, *24*), it is unexpected that we observe locally longitudinal spin in only one description of the dipole field but not the other.

The occurrence of longitudinal spin in the evanescent part of the VAS has direct consequences for a variety of experimental scenarios. In particular, whenever linear dipole emitters like atoms, quantum dots, molecules, and nanoantennas are coupled to waveguides or an optically denser medium, they might emit circularly polarized photons into defined angular regions, depending on the orientation of the dipole moment.

### Experimental verification of the longitudinal spin

**Fig. 2:**
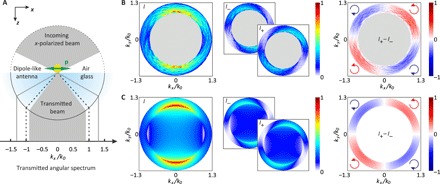
Experimental demonstration of spin-polarized light emitted by a linearly polarized dipole. (**A**) Sketch of the experimental scheme. (**B**) Experimentally measured distributions of the far-field intensity *I*, left- and right-handed circularly polarized intensities *I*_−_ and *I*_+_, and the longitudinal far-field spin density *s_r_* ∝ *I*_+_ − *I*_−_ (the handedness of the circular polarization is indicated by the red and blue arrows). All quantities are normalized to the maximum value of *I*. (**C**) Theoretically calculated distributions of *I*, *I*_−_, *I*_+_, and *s_r_*.

For an experimental demonstration, we use a dipole-like spherical plasmonic antenna positioned on a glass substrate. The interface between the air and glass half-spaces represents the *x*-*y* plane, with the surface normal ${\hat{e}}_{z}$ pointing into the glass substrate (Fig. 2A). The antenna is excited by a focused *x*-polarized beam of light, which induces a dipole moment parallel to the *x* axis. Because the glass substrate has a higher refractive index than the surrounding medium, a part of the evanescent VAS of the dipole can couple to propagating waves in the optically denser glass (*25*, *26*). An oil immersion microscope objective index-matched to the glass substrate collects the emitted VAS of the dipole up to ∣*k*_⊥_∣/*k*_0_ = 1.3. Because the VAS of the excitation beam is limited by the numerical aperture (NA) of the focusing objective (∣*k*_⊥_∣/*k*_0_ ≤ 0.9; see gray area in Fig. 2A), we can be sure that we measure only the scattered light within the angular range defined by 0.9 < ∣*k*_⊥_∣/*k*_0_ < 1.3. The experimental results are presented in Fig. 2B, where we show, from left to right, the distributions of the total intensity *I*, the left- and right-handed circularly polarized intensity components *I*_−_ and *I*_+_, and the longitudinal spin density (pointing in radial direction), here defined by *s_r_* ∝ *I*_+_ − *I*_−_. All four experimental distributions show a very good overlap with their theoretical counterparts (Fig. 2C). A derivation of the theoretical far-field distributions similar to (*25*–*28*) can be found in the Supplementary Materials. In particular, the spin density shows the expected four-lobe pattern similar to the evanescent VAS in Fig. 1B. We conclude that linearly polarized dipoles emit circularly polarized light in the evanescent part of the VAS. This notion can be crucial, when the dipole emitter is coupled to a waveguide by evanescent waves.

### Coupling of circular polarization to waveguides

**Fig. 3:**
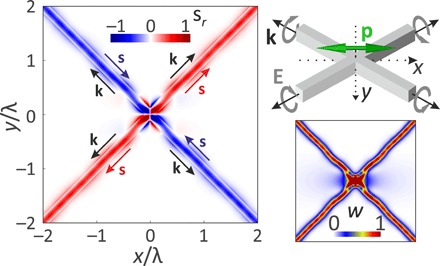
Numerically calculated spin-polarized coupling of a linearly polarized dipole to crossing waveguides. The geometry of the investigated system is depicted in the top-right inset, with the waveguide crossing in gray, the orientation of the electric dipole moment as green arrow, the propagation direction in each waveguide arm as black arrow, and the circular polarization states as gray arrows. The radial component of the spin density *s_r_* is shown for an *x*-*y* cross section. The corresponding electric field intensity *w* is shown as bottom-right inset. Both distributions are normalized to the same value.

As an example, we consider the crossing of two high–refractive index waveguides (*n* = 4) with quadratic cross sections and edge lengths of 100 nm. On top of the crossing—20 nm above the waveguides—we place a linear dipole emitter (λ = 600 nm), whereby the orientation of the dipole along the *x* axis represents the angle bisector between the waveguides. The geometry of the system is depicted in the top-right inset of Fig. 3, where we furthermore plot the numerically calculated radial component of the spin density, $s_{r}=s{\hat{e}}_{r}$, and the electric field intensity *w* = ∣**E**∣^2^ (bottom-right inset) in the *x*-*y* cross section.

Once more, we observe the coupling of elliptically polarized evanescent waves, in this case, to the waveguide modes propagating along the ±45° directions, which verifies the occurrence of longitudinal spin in the VAS of a linear dipole. The degree of circular polarization can be calculated by normalizing the spin density with the field intensity (*29*). In the center of the waveguides and far away from the dipole moment, we obtain ∣*s_r_*∣/*w* ≈ 55%. Our results therefore indicate the possibility to control the circular polarization of the guided mode with the orientation of the dipole. For example, switching from a dipole moment along the *x* direction to a dipole in the *y* direction also changes the sign of the spin in the coupled modes. Further examples can be found in the Supplementary Materials, where we also optimize the dipole moment orientation to achieve ∣*s_r_*∣/*w* ≈ 85%.

## DISCUSSION

Our results show a novel phenomenon in the light field of the most fundamental source of electromagnetic radiation—a linearly oscillating electric dipole.

At first, we establish the occurrence of an elliptically polarized electric field in the near field of a linear dipole. This notion suggests that linear dipoles may induce circularly polarized dipole moments in neighboring objects (*30*) or exert spin-dependent forces and torques on them (*31*). Nevertheless, the chirality density of the electromagnetic field of a linear dipole, as defined in (*32*), is plain zero, in particular in the far-field, where the emitted light is purely linearly polarized (*22*).

Second, we show that this situation changes for a linear dipole in the vicinity of an optically denser medium. The evanescent part of its VAS contains circularly polarized and, therefore, chiral components (*32*). These evanescent waves of the dipole are converted to circularly polarized propagating waves in the medium. Understanding of this phenomenon may enable conceptually new functionalities in future nanophotonic devices (*9*, *19*, *20*, *33*), for instance, by achieving deterministic coupling of chiral topological edge modes (*34*) and linear dipole emitters.

In this regard, it is instructive to compare the effect presented here to the directional coupling of circular dipoles to spin-momentum–locked guided modes (*6*–*10*, *12*, *35*). The spin-momentum locking links the sign of the transverse spin of an evanescent wave to the direction of its flow (*15*, *16*, *21*). Thus, the handedness of a circular dipole—or the sign of its spin—governs the direction in which it launches evanescent waves (*6*–*10*, *12*, *35*). In contrast, here, we relate the linear polarization of the dipole with the handedness of the circular polarization of the excited guided mode. This does not result in any directionality. Thus, both effects are complementary, and their combination may inspire novel approaches for spin-based on-chip optical information processing (*8*–*10*, *36*).

In conclusion, we have shown that the evanescent part of the VAS of an ideal linear dipole exhibits longitudinal spin. Evanescent waves can couple to propagating waves in a nearby optically denser medium, and we used this phenomenon to directly measure circularly polarized light emitted by a linear dipole. The presented observations shed new light on the emission of chiral photons in the complete absence of chirality in the system (*5*, *37*, *38*). We therefore expect our results to have practical implications for various classical and quantum studies even beyond nanophotonics.

*Note added in proof*: We have recently became aware of work by Joos *et al.* (*39*), exploiting coupling of a linear dipole to optical nanofibers to excite a quasicircularly polarized combination of spin-momentum locked modes.

## MATERIALS AND METHODS

### Decomposition of the spin density

For the decomposition of $\tilde{s}$ into transverse and longitudinal components (we consider the near-field part of the VAS only), we use the propagation direction vector ${\hat{e}}_{k}=k_{⊥}/|k_{⊥}|$ and the auxiliary vector ${\hat{e}}_{z}=(0,0,1)$, which is transverse with respect to the propagation direction, ${\hat{e}}_{k}\cdot {\hat{e}}_{z}=0$. A second transverse direction ${\hat{e}}_{t}$ can be constructed accordingly, ${\hat{e}}_{t}={\hat{e}}_{z}\times {\hat{e}}_{k}$. The projection onto both transverse directions results in

$${{\tilde{s}}_{z}|}_{|k_{⊥}|>k_{0}}={\tilde{s}\hat{e}}_{z}=0$$ (5)

$${{\tilde{s}}_{t}|}_{|k_{⊥}|>k_{0}}={\tilde{s}\hat{e}}_{t}\propto -\frac{2k_{x}^{2}|k_{z}|}{k_{0}^{2}|k_{⊥}|}$$ (6)

Within the chosen geometry, we observe only one transverse spin density component, which corresponds to the well-understood transverse spin of evanescent waves (*15*, *17*–*20*). The projection of the spin density vector onto the propagation direction results in

$${{\tilde{s}}_{k}|}_{|k_{⊥}|>k_{0}}=\tilde{s}\frac{k_{⊥}}{|k_{⊥}|}\propto \frac{2k_{x}k_{y}}{|k_{z}||k_{⊥}|}$$ (7)

Depending on the quadrant in *k* space, the longitudinal spin is antiparallel or parallel with respect to the propagation direction of the wave, resulting in a four-lobe structure.

### Experimental materials

An incoming linearly polarized Gaussian beam with wavelength λ = 530 nm (Δλ ≈ 1 nm) was focused by an objective with an NA of 0.9 (Leica: HCX PL FLUOTAR 100×/0.90). The tightly focused beam impinges onto the plasmonic dipole-like optical antenna. We used a gold sphere with radius *r* = 40 nm sitting on a BK7-glass substrate. The light scattered into the glass substrate was partially collected by an oil immersion–type objective with an NA of 1.3 (Leica: HCX PL FLUOTAR 100×/1.30). Behind the objective, we performed the polarization analysis using an achromatic quarter-wave plate (B. Halle Nachfl.: RAC 3.4.15) and a linear polarizer (Thorlabs: LPVISC050-MP2). Last, the back focal plane of the second objective was imaged onto a camera (The Imaging Source: DMK 23G618). For each projection onto right- and left-handed circular polarization, a back focal plane image was taken (see Fig. 2B).

## Supplementary Material

http://advances.sciencemag.org/cgi/content/full/5/6/eaav7588/DC1

Download PDF

## SUPPLEMENTARY MATERIALS

Supplementary material for this article is available at http://advances.sciencemag.org/cgi/content/full/5/6/eaav7588/DC1 Supplement 1: Real-space spin distribution. Supplement 2: Angular spectrum of a dipole. Supplement 3: Additional finite difference time-domain simulations. Fig. S1. Geometrical origin of the longitudinal spin in *k* space. Fig. S2. Numerically calculated spin-polarized waveguide coupling of linearly polarized dipoles with different dipole moment orientations. Reference (*40*)
