# Supplementary material for: Emission of circularly polarized light by a linear dipole
Source: Sci Adv. 2019 Jun 28;5(6):eaav7588. doi: 10.1126/sciadv.aav7588 (PMC6598770; doi:10.1126/sciadv.aav7588)
Supplement: http://advances.sciencemag.org/cgi/content/full/5/6/eaav7588/DC1 [file supp_5_6_eaav7588__index.html]

Science Advances | Science Advances

## Supplementary Materials

**This PDF file includes:**

- Supplement 1: Real-space spin distribution.
- Supplement 2: Angular spectrum of a dipole.
- Supplement 3: Additional finite difference time-domain simulations.
- Fig. S1. Geometrical origin of the longitudinal spin in *k* space.
- Fig. S2. Numerically calculated spin-polarized waveguide coupling of linearly polarized dipoles with different dipole moment orientations.
- Reference (*40*)

Download PDF

**Files in this Data Supplement:**

- Adobe PDF - aav7588\_SM.pdf
