# Supplementary material for: Emission of circularly polarized light by a linear dipole
Source: Sci Adv. 2019 Jun 28;5(6):eaav7588. doi: 10.1126/sciadv.aav7588 (PMC6598770; doi:10.1126/sciadv.aav7588)
Supplement: Download PDF [file aav7588_SM.pdf]

Science Advances
AAAS

advances.sciencemag.org/cgi/content/full/5/6/eaav7588/DC1

Supplementary Materials for

Emission of circularly polarized light by a linear dipole

Martin Neugebauer, Peter Banzer, Sergey Nechayev*

*Corresponding author. Email: sergey.nechayev@mpl.mpg.de

Published 28 June 2019, *Sci. Adv.* **5**, eaav7588 (2019)
DOI: 10.1126/sciadv.aav7588

This PDF file includes:

Supplement 1: Real-space spin distribution.
Supplement 2: Angular spectrum of a dipole.
Supplement 3: Additional finite difference time-domain simulations.
Fig. S1. Geometrical origin of the longitudinal spin in *k* space.
Fig. S2. Numerically calculated spin-polarized waveguide coupling of linearly polarized dipoles with different dipole moment orientations.
Reference (*40*)

Supplement 1: Real-space spin distribution.

In order to calculate the spin density distribution surrounding a linear dipole emitter with dipole moment $\mathbf{p}$, we rewrite Eq. 1 of the manuscript using the vectors $\mathbf{u} = (\hat{\mathbf{e}}_\mathbf{r} \times \mathbf{p}) \times \hat{\mathbf{e}}_\mathbf{r}$ and $\mathbf{v} = 3\hat{\mathbf{e}}_\mathbf{r} (\hat{\mathbf{e}}_\mathbf{r} \cdot \mathbf{p}) - \mathbf{p}$ with $\hat{\mathbf{e}}_r = (x, y, z) / r$

$$\mathbf{E}(\mathbf{r}) = \frac{e^{\imath k_0 r}}{4\pi\varepsilon_0} \left(\frac{k_0^2}{r}\mathbf{u} + \frac{1}{r^3}\mathbf{v} - \frac{\imath k_0}{r^2}\mathbf{v} \right) \tag{S1}$$

In the chosen representation, the three terms within the brackets dominate the far-field ($\propto 1/r$), the near field ($\propto 1/r^3$), and the intermediate field ($\propto 1/r^2$), respectively (*22*). Since the far-field and the near-field terms are in-phase (both terms are real), their vectorial interference cannot result in elliptical polarization. The same holds true for the interference of the near and intermediate fields, since they are parallel (both of them are pointing in the direction of $\mathbf{v}$). This leaves only the interference of the terms describing the far field and the intermediate field to be the cause of the real-space transverse spin density distribution plotted in Fig. 1C of the manuscript, which can be calculated by introducing Eq. S1 in the definition of the electric spin density distribution in real space, where we omit all prefactors for the sake of simplicity (Eq. 4 in the manuscript)

$$\mathbf{s}(\mathbf{r}) = \mathrm{Im}(\mathbf{E}^* \times \mathbf{E}) \propto -\frac{k_0^3}{r^3}\mathbf{u} \times \mathbf{v} = \frac{2k_0^3}{r^3} (\hat{\mathbf{e}}_r \cdot \mathbf{p}) \hat{\mathbf{e}}_r \times \mathbf{p} \tag{S2}$$

The vector product indicates that the spin density is purely azimuthal around the axis of the dipole moment. For a dipole moment parallel to the x-axis, with $\mathbf{p} = (p_0, 0, 0)$, we result in

$$\mathbf{s}(\mathbf{r}) \propto \frac{k^3 p_0^2}{r^5} \begin{pmatrix} 0 \\ xz \\ -xy \end{pmatrix} \tag{S3}$$

Here, the spin density is azimuthal around the x-axis, and it changes its sign depending on the half-space ($x \gtrless 0$). In the manuscript this sign change is indicated by the color coding of the cones (red and blue) in Fig. 1C.

It should be mentioned that in recent works, spinning electric fields have been observed in the vicinity of achiral sub-wavelength scatterers excited by linearly polarized light. These studies, however, explain the occurrence of spin by interference of the dipole field with the excitation field (*30*), or with higher order modes (*38,40*). In contrast, the transverse spin $\mathrm{s}(\mathbf{r}) \propto \frac{xz}{r^5}\hat{\mathbf{e}}_y - \frac{xy}{r^5}\hat{\mathbf{e}}_z$ in Fig. 1C arises in the vicinity of a point-dipole (without an excitation field) as a result of the interference of the first and the third term of the dipole field in Eq. 1.

Supplement 2: Angular spectrum of a dipole.

2.1 Dipole in free space

The following calculation closely follows the formalism utilized in ref. (*28*). We begin with the field distribution of an electric dipole emitter, which exhibits an arbitrarily oriented dipole moment $\mathbf{p}$. The dipole is placed at the origin of a Cartesian coordinate frame. Utilizing the

Green's function description, the emitted electric field can be calculated by (28)

$$\mathbf{E}\left(\mathbf{r}\right)=\omega^{2}\mu_{0}\widehat{\mathbf{G}}\left(\mathbf{r}\right)\mathbf{p} \tag{S4}$$

where ω and μ_0 are the angular frequency of the emitted light and the magnetic permeability of the vacuum. The Green's function tensor $\widehat{\mathbf{G}}$ in the half-space ($z > 0$) is defined by (28)

$$\widehat{\mathbf{G}}\left(\mathbf{r}\right)=\frac{\imath}{8\pi^{2}}\iint_{-\infty}^{\infty}\frac{1}{k_{z}}\widehat{\mathbf{M}}e^{\imath\left(k_{x}x+k_{y}y+k_{z}z\right)}dk_{x}dk_{y} \tag{S5}$$

$$\widehat{\mathbf{M}}=\begin{pmatrix}1-\frac{k_{x}^{2}}{k_{0}^{2}} & -\frac{k_{x}k_{y}}{k_{0}^{2}} & -\frac{k_{x}k_{z}}{k_{0}^{2}}\\ -\frac{k_{x}k_{y}}{k_{0}^{2}} & 1-\frac{k_{y}^{2}}{k_{0}^{2}} & -\frac{k_{y}k_{z}}{k_{0}^{2}}\\ -\frac{k_{x}k_{z}}{k_{0}^{2}} & -\frac{k_{y}k_{z}}{k_{0}^{2}} & 1-\frac{k_{z}^{2}}{k_{0}^{2}}\end{pmatrix} \tag{S6}$$

For an x-polarized dipole, $\mathbf{p}=(p_0,0,0)$, only the first column of matrix $\widehat{\mathbf{M}}$ is relevant, and we can write

$$\mathbf{E}\left(\mathbf{r}\right)\propto\iint_{-\infty}^{\infty}\frac{1}{k_{z}}\begin{pmatrix}1-\frac{k_{x}^{2}}{k_{0}^{2}}\\ -\frac{k_{x}k_{y}}{k_{0}^{2}}\\ -\frac{k_{x}k_{z}}{k_{0}^{2}}\end{pmatrix}e^{\imath\left(k_{x}x+k_{y}y+k_{z}z\right)}dk_{x}dk_{y}\propto\iint_{-\infty}^{\infty}\widetilde{\mathbf{E}}\left(\mathbf{k}\right)e^{\imath\mathbf{kr}}dk_{x}dk_{y} \tag{S7}$$

As we can see, we can associate $\widetilde{\mathbf{E}}\left(\mathbf{k}\right)$ as defined in the manuscript, with the vectorial angular spectrum (VAS) of the dipole emitter. Calculating the spin density distribution of the VAS yields

$$\widetilde{\mathbf{s}}\left(\mathbf{k}\right)=\operatorname{Im}\left(\widetilde{\mathbf{E}}^{*}\times\widetilde{\mathbf{E}}\right)\propto\begin{pmatrix}2k_{x}^{2}k_{y}\operatorname{Im}\left(\frac{1}{k^{4}k_{z}}\right)\\ 2k_{x}\operatorname{Im}\left(\frac{1}{k^{2}k_{z}}\right)-2k_{x}^{3}\operatorname{Im}\left(\frac{1}{k^{4}k_{z}}\right)\\ 0\end{pmatrix} \tag{S8}$$

The projections of $\widetilde{\mathbf{s}}$ onto $\operatorname{Re}\left(\mathbf{k}\right)=\mathbf{k}_{\perp}$ and $\hat{\mathbf{e}}_z\times\mathbf{k}_{\perp}$ result in the longitudinal and transverse spin density components (see manuscript), respectively. The spin density component parallel to $\hat{\mathbf{e}}_z$ is zero.

2.2 Dipole on interface

In order to calculate the far field of an x-polarized dipole sitting on a glass substrate (we only consider the emission into the optically denser glass), we need to transform the polarization

vector of the VAS into the p- and s-polarization basis of the air-glass-interface. This is achieved by using a complex-valued 3×2 rotation matrix projecting Cartesian coordinates $(\mathbf{e}_x, \mathbf{e}_y, \mathbf{e}_z)$ onto the eigenbasis of the system $(\mathbf{e}_p, \mathbf{e}_s)$

$$\widehat{\mathbf{R}} = \begin{pmatrix} \cos(\phi)\cos(\theta) & \sin(\phi)\cos(\theta) & -\sin(\theta) \\ -\sin(\phi) & \cos(\phi) & 0 \end{pmatrix} \tag{S9}$$

With k-space coordinates and the definitions, $\sin(\theta) = k_\perp/k$, $\cos(\theta) = k_z/k$, $\sin(\phi) = k_y/k_\perp$, $\cos(\phi) = k_x/k_\perp$ and $k_\perp = |\mathbf{k}_\perp|$, the matrix can be simplified to

$$\widehat{\mathbf{R}} = \begin{pmatrix} \frac{k_x k_z}{k_\perp k_0} & \frac{k_y k_z}{k_\perp k_0} & -\frac{k_\perp}{k_0} \\ -\frac{k_y}{k_\perp} & \frac{k_x}{k_\perp} & 0 \end{pmatrix} \tag{S10}$$

Multiplication of $\widehat{\mathbf{R}}$ and $\widetilde{\mathbf{E}}(\mathbf{k})$ results in

$$\widetilde{\mathbf{E}}'(\mathbf{k}) \propto \frac{1}{k_z} \begin{pmatrix} \frac{k_x k_z}{k_\perp k_0} \\ -\frac{k_y}{k_\perp} \end{pmatrix} \tag{S11}$$

As a next step, we adapt Eq. S11 in order to describe the influence of an air-glass-interface, where $z < 0$ ($z > 0$) corresponds to the air (glass) half-space. In particular, we consider the dipole to be in air with distance d to the interface. We choose the projection of the dipole onto the interface as the origin of the Cartesian coordinate frame, which requires us to multiply $\widetilde{\mathbf{E}}'$ with the propagator $e^{\imath k_z d}$. In order to calculate the transmission through the interface, we multiply the individual field components E'_p and E'_s with the Fresnel transmission coefficients (*28*)

$$t_p = \frac{2 n k_z}{n^2 k_z + \sqrt{k_0^2 n^2 - k_\perp^2}} \tag{S12}$$

$$t_s = \frac{2 k_z}{k_z + \sqrt{k_0^2 n^2 - k_\perp^2}} \tag{S13}$$

To transform the transmitted VAS into the far field, we need to multiply with a factor $\propto k_z^{\text{glass}} = \sqrt{k_0^2 n^2 - k_\perp^2}$. Finally, the far-field in the glass half-space reads

$$\widetilde{\mathbf{E}}_f(\mathbf{k}) \propto e^{\imath k_z d} \frac{\sqrt{k_0^2 n^2 - k_\perp^2}}{k_z} \begin{pmatrix} t_p \frac{k_x k_z}{k_\perp k_0} \\ -t_s \frac{k_y}{k_\perp} \end{pmatrix} \tag{S14}$$

A straight forward calculation of the far-field spin density above the critical angle ($k_\perp > k_0$) results in

$$\widetilde{s}_r\big|_{|\mathbf{k}_\perp|>k_0} \propto I_+ - I_- \propto \mathrm{Im}\left(\widetilde{E}^*_{f,s}\widetilde{E}_{f,p}\right) \propto e^{-2|k_z|d}\frac{k_0^2 n^2 - k_\perp^2}{k_0 k_\perp}\,\mathrm{Re}\left(t_p^* t_s\right)\,\widetilde{s}_k\big|_{|\mathbf{k}_\perp|>k_0} \tag{S15}$$

with the longitudinal component of the spin density of the VAS, $\widetilde{s}_k\big|_{|\mathbf{k}_\perp|>k_0} \propto 2k_x k_y/\left(|k_z|\,k_\perp\right)$, as defined by Eq. 7 in the manuscript. We see that the longitudinal spin of the VAS of a linear dipole propagates to the far-field, when the dipole is close to an optically denser medium ($d \ll \lambda$). The amplitude of the far-field longitudinal spin only differs from the longitudinal spin of the VAS by a geometric factor and the Fresnel coefficients. For the theoretical images in Fig. 2C of the manuscript, an additional energy conservation factor $\propto \left(k_0^2 n^2 - k_\perp^2\right)^{-1/2}$ is included, taking into account the aplanatic oil immersion microscope objective used for collecting the scattered light (*28*).

2.3 Geometrical origin of the longitudinal spin

In order to provide an intuitive picture explaining the occurrence of the longitudinal spin and its four-lobe pattern in the near-field part of the VAS, we deploy a simplified geometrical model starting with the sketch in fig. S1A. It shows the x-polarized dipole moment $\mathbf{p}$ (green vector) in the original Cartesian coordinate system (x, y, z). Now we consider the coupling of the dipole to evanescent waves propagating in x-direction ($k_y = 0$, $k_x > k_0$, and $k_z = \imath\,|k_z|$). In principle, a dipole can couple to two different polarization states of the electric field, one being parallel to the x-z-plane—transverse magnetic (TM)—and one perpendicular to said plane—transverse electric (TE). For an exemplary point in time, a side view sketch of the electric field distributions of the evanescent waves is depicted in fig. S1B. The TM evanescent wave exhibits the well-known transversely spinning electric field vector as indicated in the upper graph (*15-21*). In other words, the longitudinal field E_x and the transverse field E_z have a relative phase of $\pi/2$. In contrast, the electric field of the TE evanescent wave (E_y) points in and out of the

plane (see lower graph). As we can see, only the electric field of the TM wave—in particular its x component—can overlap with the dipole moment, which implies that the dipole emits purely TM evanescent waves along the x-axis. In this case, the spin is purely transverse (the electric field is spinning in the meridional x-z-plane) and no longitudinal spin occurs, as can be seen in the projection of the polarization ellipse onto the y-z-plane in fig. S1C.

However, when we consider the emission along a different direction $\acute{x}$ in a rotated Cartesian coordinate system ($\acute{x}$, $\acute{y}$, $\acute{z}$), we see that the dipole can couple to TE evanescent waves as well, because, with respect to the new meridional $\acute{x}$-$\acute{z}$-plane, the dipole exhibits an additional out-of-plane $p_{\acute{y}}$ component. This results in a superposition of TE and TM polarized evanescent waves. The additional TE field tilts the polarization ellipse of the TM evanescent wave out of the meridional $\acute{x}$-$\acute{z}$-plane. The tilt finally leads to the occurrence of longitudinal spin, this is a field vector spinning around the propagation axis. For illustration, we plot the projections of the polarization ellipse onto the $\acute{y}$-$\acute{z}$-plane (see fig. S1C). The sign of the longitudinal spin depends on the relative phase between TE and TM polarized evanescent waves, which itself depends on the projection of $\mathbf{p}$ onto $\acute{\mathbf{x}}$ and $\acute{\mathbf{y}}$. Therefore, the relative phase between TE and TM polarized evanescent waves alternates between 0 and π for the corresponding quadrants, leading to the four-lobe patterns of the longitudinal spin in Figs. 1B,2B,3 of the manuscript.

Supplement 3: Additional finite difference time-domain simulations.

Here, we discuss the results of five additional finite difference time domain simulations (all numerical calculations where performed using FDTD Solutions from Lumerical Solutions Inc.), in order to investigate the influence of the orientation of the linear dipole moment on the waveguide coupling. In fig. S2A, we again plot the radial component of the spin density s_r and the electric field intensity $w = |E|^2$ for a dipole oriented along the x-axis (see also Fig. 3 of the manuscript). For comparison, we depict the same parameters for a dipole oriented along

the y-axis (fig. S2B), oriented along the $45\,°$-direction (fig. S2C), and oriented along the z-axis (fig. S2D), this is perpendicular to the plane of observation. The geometry of the system including the waveguide crossing is indicated by the corresponding insets. We see that the orientation of the dipole strongly influences the coupling. As mentioned in the manuscript, the rotation of the dipole moment by $90°$ around the z-axis (dipole moment parallel to the y-direction) changes the sign of the coupled spin (fig. S2B). This proves that the orientation of linearly polarized dipoles can be used for controlling circular polarization and spin multiplexing at the nanoscale. No spin is coupled when the dipole moment is parallel/perpendicular to the waveguides (fig. S2C) or out-of-plane (fig. S2D). The oscillating spin density distributions in the outer areas of the waveguides are a result of the interference of p-polarized and s-polarized first and second order modes, which propagate with different effective k-vectors.

As a next step, we optimize the degree of circularly polarized light coupled to the waveguides. In the presented system, the best result can be achieved for a dipole being rotated by $\approx 25°$ around the z-axis (fig. S2E). Here, the spin is coupled into the two waveguides with different efficiencies. In $-45°$-direction we couple $\approx 85\%$ of circular polarization, however at the cost of a reduced field intensity with respect to the coupling in $+45°$-direction, where $|s_r|/w \approx 22\%$.

Finally, we demonstrate that the longitudinal spin of the VAS of a linear dipole also couples to a single waveguide geometry (fig. S2F). The results show that the observed effect is not caused by the junction of both waveguides, verifying that the spin-polarized coupling is in fact linked to the the VAS.

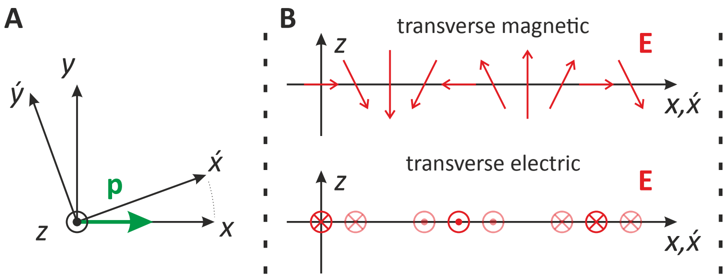

Fig. S1. Geometrical origin of the longitudinal spin in k space. (**A**) Sketch of the Cartesian coordinate systems and the dipole moment. (**B**) Side view sketch of transverse magnetic and transverse electric polarized evanescent waves. (**C**) Projections of the polarization ellipses onto the transverse plane.

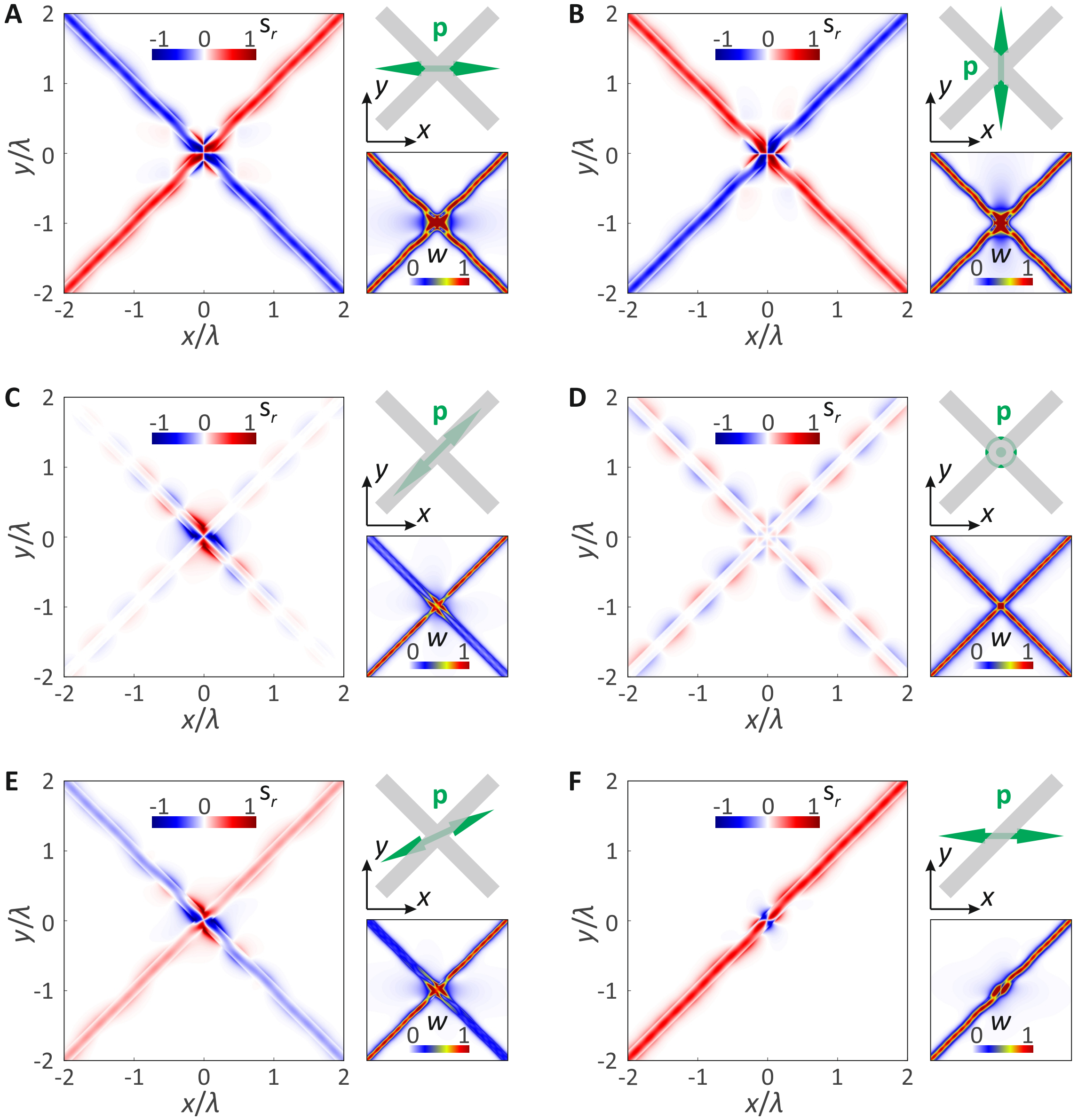

Fig. S2. Numerically calculated spin-polarized waveguide coupling of linearly polarized dipoles with different dipole moment orientations. (**A**-**E**) The geometry of the investigated system is depicted in the upper right inset, with the waveguide crossing in gray and the orientation of the electric dipole moment (in this representation placed behind the waveguide crossing) depicted as green arrow. The radial component of the spin density s_r is shown for an x-y-cross-section. The corresponding electric field intensity w is shown as lower right inset. Both distributions are normalized to the same value. (**F**) The spin-polarized coupling to a single waveguide is shown similar to **A**-**E**.
